# Profile of Nucleotides in Chinese Mature Breast Milk from Six Regions

**DOI:** 10.3390/nu14071418

**Published:** 2022-03-29

**Authors:** Lutong Yang, Zhiheng Guo, Miao Yu, Xiaokun Cai, Yingyi Mao, Fang Tian, Wenhui Xu, Guoliang Liu, Xiang Li, Yanrong Zhao, Lin Xie

**Affiliations:** 1 Department of Nutrition and Food Hygiene, School of Public Health, Jilin University, Changchun 130021, China; yanglt18@mails.jlu.edu.cn (L.Y.); ymiao17@mails.jlu.edu.cn (M.Y.); xuwh20@mails.jlu.edu.cn (W.X.); glliu@jlu.edu.cn (G.L.); 2 Department of Obstetrics, First Hospital, Jilin University, Changchun 130021, China; zhihengguo277@jlu.edu.cn; 3 Abbott Nutrition Research and Development (R&D) Centre, Shanghai 200233, China; xiaokun.cai@abbott.com (X.C.); yingyi.mao@abbott.com (Y.M.); fang.tian@abbott.com (F.T.); xiang.li@abbott.com (X.L.)

**Keywords:** MUAI, total potentially available nucleosides, breast milk, nucleotides

## Abstract

This study measured the total potentially available nucleoside (TPAN) content in breast milk from six different regions of China as a part of the Maternal Nutrition and Infant Investigation (MUAI) study. A total of 631 breast milk samples were collected from healthy, lactating women with singleton, full-term pregnancies between 40 and 45 days postpartum in Changchun, Chengdu, Lanzhou, Shanghai, Tianjin, and Guangzhou. TPAN and free 5′-monophosphate nucleotide (5′-MNT) contents were determined by high-performance liquid chromatography. The TPAN content of the Chinese mature milk ranged from 11.61 mg/L to 111.09 mg/L, with a median level of 43.26 mg/L. Four types of nucleotides were identified, and the median levels of cytidine monophosphate (CMP), uridine monophosphate (UMP), guanosine monophosphate (GMP), and adenosine monophosphate (AMP) were 22.84 mg/L, 9.37 mg/L, 4.86 mg/L, and 4.80 mg/L, respectively. CMP was the predominant nucleotide, accounting for 52.9% of the TPAN content, while free 5′-MNT accounted for 18.38% of the TPAN content. The distribution pattern of the TPAN content and level of the individual nucleotides were significantly different among the selected regions (*p* < 0.05), but the result showed no significant differences in the TPAN level in breast milk (*p* > 0.05). In addition, no correlation was reported between the geographic distribution and TPAN levels. This result showed that TPAN better reflects the level of total potential nucleosides in Chinese breast milk rather than 5′-MNT in free form. CMP, UMP, GMP, and AMP are the only 4 types of nucleotides reported in all detections. In addition, results revealed a large variation of TPAN levels in Chinese breast milk across six regions, so that the median value may not be the optimal fortification level of TPAN for Chinese infant populations.

## 1. Introduction

Breast milk is the optimal food for infants, as it provides essential nutrition, antibodies, and other important nutrients to support the growth and development of infants [1,2,3]. Nitrogen in breast milk consists of up to 20% non-protein nitrogen, which plays a crucial role in infant growth [4,5,6]. Nucleotides, 2–5% of non-protein nitrogen, comprises a nitrogenous base, a sugar moiety, and phosphate groups, which are involved in many biological processes and play a vital role in the utilization of protein in breastfed infants [6,7,8]. In addition, nucleotides, as the monomeric units of ribonucleic acid (RNA) and deoxyribonucleic acid (DNA), are important in encoding genetic information, regulating energy metabolism, and signal transduction, as well as supporting rapid growth in early infancy [9]. Nucleotides can be synthesized in the human body through the following two pathways: (1) de novo synthesis using glutamine, aspartic acid, carbon dioxide, and other substances, and (2) through the salvage pathway using metabolized bases and nucleosides hydrolyzed from the diet [10,11]. Nucleotides can be synthesized endogenously, but infants show a limited capacity to synthesize the endogenous nucleotides needed to meet their increased metabolic demands for growth and development. Consequently, nucleotides are considered conditionally-essential nutrients, becoming essential during infancy, particularly in the gastrointestinal and immune systems [9]. Therefore, dietary nucleotides are important for infants and are consumed from either breast milk or infant formulas. 

Breast milk contains different sources of nucleotides, including free nucleosides (nucleosides are components of nucleic acids and nucleotides. Nucleosides are formed by the condensation of ribose or deoxyribose with nitrogenous bases) and nucleotides, nucleoside-containing adducts (such as nicotinamide-adenine dinucleotide and uridine-diphosphate glucose), and nucleoside polymers (such as RNA) [12,13]. Nucleotide polymers and adducts can be converted into nucleotides by proteases and nucleases, and nucleotides can be further converted into nucleosides by intestinal alkaline phosphatase and nucleotidases [6,14]. The compounds obtained from the degradation of different sources of nucleotides can be further utilized in the liver cells [15]. Compared with cow’s milk, human milk contains more non-protein nitrogen compounds, such as nucleotides and nucleosides [16,17]. Thus, cow’s milk-based infant formula should ideally be designed based on human milk composition to meet infants’ needs. In most countries, monophosphate nucleotides are only allowable in fortification form in infant formula to mimic the composition of ‘gold-standard’ human milk [18,19]. However, the addition standards vary among different countries. Breast milk has largely been investigated to elucidate its unique nucleotide composition. However, most published studies have only detected the monophosphate nucleotides that are the main form of nucleotide fortification in infant formula, while some studies also considered the free nucleotides and nucleosides [8,20,21]. These studies analyzed partial sources of nucleotides in breast milk and showed markedly variable results due to the different pretreatment and assessment methods. Only two studies have measured all of the major sources of nucleotides in breast milk, but they used mixed populations [22,23]. The total nucleotides in milk were expressed as the total potentially available nucleosides (TPAN) in two studies, by Leach et al. [22] and Tressler et al. [23]. The detection method in our study was modified based on Leach et al. [22], and the content of TPAN in breast milk was detected to evaluate the full content of nucleotides in Chinese breast milk.

Currently, there are limited reports on the nucleotides content in Chinese mature milk. The aims of this study were to analyze the content and composition of TPAN in breast milk, exclusively from China, and to evaluate interregional differences. In addition, associations with maternal basic demographic characteristics, such as age, pre-pregnancy body mass index (BMI), pre-delivery BMI, gestational weight gain, and gestational period, were investigated.

## 2. Materials and Methods

### 2.1. Subject Selection

A total of 631 healthy lactating mothers were recruited from Changchun, Tianjin, Lanzhou, Shanghai, Chengdu, and Guangzhou between 40 and 45 days postpartum. Written informed consent was obtained before study enrollment and sample collection. This study was a part of the Maternal Nutrition and Infant Investigation (MUAI) study. The study protocol was registered in the Chinese Clinical Trial Registry (ChiCTR) with the registration number ChiCTR1800015387. The parent study was designed to investigate the nutritional components in cord and maternal blood as well as breast milk from healthy, Chinese women across six regions of China, and to further explore the potential correlation with the dietary intake of pregnant and lactating women.

Eligible lactating women were recruited through the obstetric departments of hospitals or maternity care centers. The criteria for maternal and infant inclusion were maternal age between 20 and 35 years, and single child delivered between 37 and 42 weeks gestation with an Apgar score of >8. Participants were asked to plan a breastfeeding period of at least three months. Lactating women with metabolic diseases, severe heart diseases, acute and chronic infectious diseases, or who were taking drugs affecting the nutrient metabolism, and infants with congenital diseases or with breast-feeding contraindications were excluded.

### 2.2. Data Collection

All participants responded to a questionnaire that included the basic demographic characteristics of the mother including the following: maternal age, height, weight, pre-pregnancy weight, pre-delivery weight, gestational weight gain, gestational period, delivery mode, and educational level. Additionally, information on the sex, length, and weight of infants was also collected.

### 2.3. Sample Collection

To avoid a circadian influence on the results, breast milk samples of the lactating mothers (40–45 days postpartum) were collected during the second feeding session in the morning (9:00–11:00 a.m.). The samples were thoroughly mixed and poured into 50 mL centrifuge tubes, which were immediately wrapped in tin foil and stored at 4 °C. A courier was arranged to pick up the samples. Breast milk samples were placed in a foam insulation box with ice packs and sent to the laboratory within 2 h. Upon arrival at the laboratory, the agglomerated fat in the samples was re-melted in a 30 °C water bath and mixed for 2 min for complete homogenization. Subsequently, the samples were stored in a freezer at −80 °C.

### 2.4. Analytical Methods

#### 2.4.1. Chemicals and Reagents

Cytosine (100% purity), uracil (100% purity), guanine (99% purity), adenine (100% purity), hypoxanthine (100% purity) standards, and 5-methyl cytidine (purity ≥ 99%), 1-methylguanosine (100% purity), adenosine 5′-monophosphate sodium salt, cytidine 5′-monophosphate disodium salt, guanosine 5′-monophosphate disodium salt, uridine 5′-monophosphate disodium salt, inosine 5′-monophosphate disodium salt, 5′thymidylic acid disodium salt (purity ≥ 99%), nuclease (nuclease P1), pyrophosphatase (nucleotide pyrophosphatase), and phosphatase (bacterial alkaline phosphatase) reagents were purchased from Sigma.

#### 2.4.2. Sample Preparation

Sample preparation for detecting TPAN content was carried out as follows: Samples were rapidly thawed at room temperature and 0.5 mL of the breast milk sample was accurately pipetted into a bottle, followed by the addition of 2.5 mL of water. After adding 5-methylcytidine and 1-methylguanosine to the sample as internal standards, it was heated at 110 °C for 15 min. There are many active enzymes in breast milk, of which adenosine deaminase (ADA) can convert AMP to inosine monophosphate (IMP.) Rapid heating of the milk inactivates the majority of the interfering enzymes but does not change the content of the total nucleotides. Subsequently, the breast milk samples were incubated for 16~18 h at 37 °C with nuclease to hydrolyze the polymeric to monomeric nucleotides. This was followed by incubation with pyrophosphatase and phosphatase for 3 h at 37 °C to hydrolyze the nucleotides to nucleosides. The treated sample was finally diluted to 12 mL with 0.5 mol/L potassium dihydrogen phosphate.

Polyacrylamide gel (Affi Gel-601) was used as the solid-phase extraction medium. The Affi-Gel 601 was mixed with 0.1 mol/L potassium phosphate in a ratio of 1:100. The hydrated precipitation gel (500 µL) in a polypropylene micro-tube was washed once with 0.25 mol/L phosphoric acid and washed twice with 0.25 mol/L potassium phosphate. A 1 mL sample from the enzymatic preparation described above was added to the gel and vortexed, binding the nucleosides to the gel. Contaminating compounds were removed from the gel-bound nucleosides by washing twice with potassium dihydrogen phosphate buffer (1 mL). Nucleosides were then eluted from the gel using 1 mL 0.25 mol/L phosphoric acid and passed through a 0.2 µm filter directly into a vial for high-pressure liquid chromatography (HPLC) analysis. 

Sample preparation for detecting free 5′-monophosphate nucleotide (5′-MNT) content was carried out as follows: 0.5 mL breast milk sample was pipetted into a centrifuge tube, followed by the addition of 1.5 mL extraction solution (1 mol/L sodium chloride: 5 mmol/L ethylene diamine tetraacetic acid). After adding 0.1 mL internal standards (5′thymidylic acid disodium salt) and 0.4 mL water to the sample, the sample was vortexed vigorously for 10 s, then allowed to stand for about 10 min. A solid phase extraction column (SPE column) was placed on the vacuum manifold. A total of 4 mL methanol was added into the SPE column, followed by 2 × 5 mL water washes. Then, after adding 2 mL of the designated ample solution prepared above to the SPE column for filtration, the 5′-MNT was bound to the SPE column. Contaminating compounds were removed from the SPE column by washing with 0.3 mol/L potassium bromide (4 mL). The 5′-MNT from each SPE column was eluted by 4 mL of 10 mmol/L potassium dihydrogen phosphate/L at <2 mL/min flow rate. Finally, 5 mL syringes and 0.22 µm filter, directly into a vial, was used for high-pressure liquid chromatography (HPLC) analysis.

#### 2.4.3. HPLC Analysis

ZORBAX Eclipse XDB-C18 (4.6 mm × 150 mm, 3.5 µm) was selected as the HPLC column to detect the TPAN in the breast milk. The column temperature was 22 °C, injection volume 100 µL, tray temperature 16 °C, and the detection wavelength was 260 nm. Mobile phase A was 21.75 mmol/L ammonium acetate solution and 2 mmol/L sodium hexanesulfonate; mobile phase B was composed of mobile phase A and acetonitrile (the ratio was 100:6).

Gemini C18 (4.6 mm × 250 mm, 5 µm) was selected as the HPLC column to detect the 5′-MNT in breast milk. The column temperature was 35 °C, injection volume 50 µL, and the detection wavelength was 260 nm. Mobile phase A was 0.1 mol/L potassium phosphate monobasic; mobile phase B was mobile phase A/20% methanol.

### 2.5. Data Analysis

Data were analyzed using SPSS 26.0. Descriptive data were reported as means ± standard deviations or medians and interquartile ranges [M (P25, P75)]. The Kruskal–Wallis test was used to compare the data from different regions since the data did not meet the assumptions of normal distribution and homogeneity of variance. Nonparametric Mann–Whitney U-test was employed to detect specific differences between the six regions. Spearman and Kendall’s tau-b correlation coefficients were used to examine the relationships between the basic demographic characteristics and TPAN content of breast milk and *p* values of <0.05 were considered statistically significant.

## 3. Results

### 3.1. Characteristics of the Subjects

Overall, 631 healthy, lactating mothers were recruited in this study. All of the participants were Han Chinese, and the mean age of the participants was 29 years. The mean pre-pregnancy BMI and pre-delivery BMI of the participants were 21.1 and 26.9 kg/m^2^, respectively. The average weight gain during pregnancy was 14.60 kg. Overall, 44.4% of the participants had undergone cesarean sections and 55.6% had vaginal deliveries. Most of the lactating women had completed college education. The basic demographic characteristics of the mothers and newborns from the six regions are described in Table 1.

### 3.2. TPAN and 5′-MNT Content of Mature Milk

The median content and composition of TPAN in mature milk are shown in Table 2. The median TPAN content was 43.26 mg/L, ranging from 11.61 mg/L to 111.09 mg/L. The results showed that, depending on the nitrogenous base, nucleotides were grouped into different classes, including cytidine monophosphate (CMP), uridine monophosphate (UMP), guanosine monophosphate (GMP), and adenosine monophosphate (AMP). CMP (22.84 mg/L) was the predominant nucleotide, followed by UMP (9.37 mg/L), GMP (4.86 mg/L), and AMP (4.80 mg/L). Inosine monophosphate (IMP) could not be quantified in all of the breast milk samples (limit of quantitation = 1.04 mg/L).

**Figure 1:**
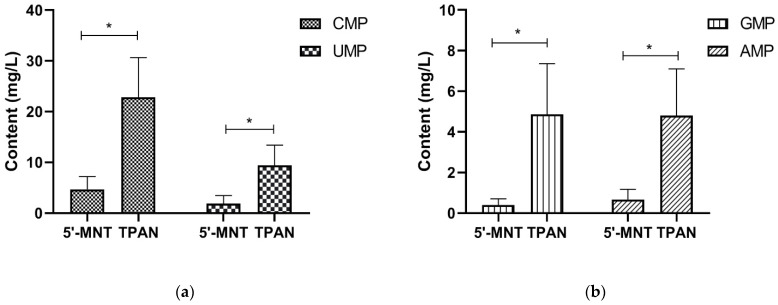
Comparison of 5′-MNT contents and TPAN contents in human milk. (**a**) Comparison of pyrimidine nucleotide contents in 5′-MNT and TPAN; (**b**) Comparison of purine nucleotide contents in 5′-MNT and TPAN. CMP, Cytidine monophosphate; UMP, uridine monophosphate; GMP, guanosine monophosphate; AMP, adenosine monophosphates; 5′-MNT, free 5′-monophosphate nucleotides; TPAN, total potentially available nucleosides. * Indicates *p* < 0.05.

**Figure 2:**
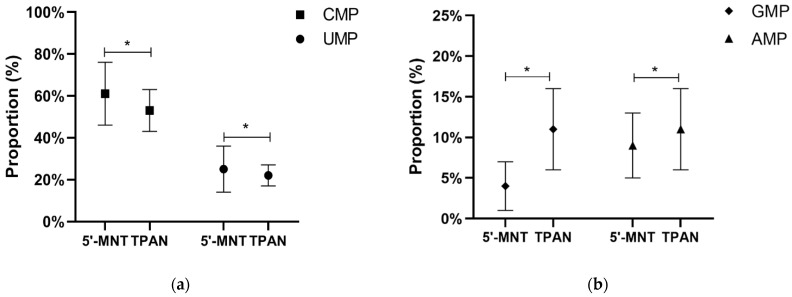
Comparison of 5′-MNT contents and TPAN contents in human milk. (**a**) Comparison of CMP, UMP proportion in 5′-MNT and TPAN; (**b**) Comparison of GMP, AMP proportion in 5′-MNT and TPAN. CMP, Cytidine monophosphate; UMP, uridine monophosphate; GMP, guanosine monophosphate; AMP, adenosine monophosphates; 5′-MNT, free 5′-monophosphate nucleotides; TPAN, total potentially available nucleosides. * Indicates *p* < 0.05.

In addition to the analysis of the TPAN content of breast milk, the 5′-MNT content of the mature milk was analyzed by HPLC. As with TPAN, four types of nucleotides were detected. The median 5′-MNT content was 7.95 mg/L, ranging from 0.90 mg/L to 25.35 mg/L (Table 2). It can be seen from Figure 1 that the content of the four types of nucleotides in TPAN was significantly higher than in 5′-MNT (*p* < 0.05). The 5′-MNT content accounted for 18.38% of the total TPAN content. The statistical analysis showed that the nucleotide composition of TPAN was significantly different from that of 5′-MNT (*p* < 0.05, Figure 2). Therefore, TPAN content was not only higher than that of 5′-MNT, but also the nucleotide composition of TPAN was different from that of 5′-MNT.

### 3.3. TPAN Content of Mature Milk from the Six Regions

The TPAN content of the mature milk from the six different regions is shown in Table 3. The contents of TPAN in the mature milk from Changchun, Chengdu, Lanzhou, Shanghai, Tianjin, and Guangzhou were 47.01 mg/L, 42.58 mg/L, 45.36 mg/L, 43.83 mg/L, 41.02 mg/L, 42.12 mg/L, respectively. However, the statistical analysis showed no significant difference in the UMP and TPAN content of the milk from the different regions (*p* > 0.05). The CMP level of the milk from Lanzhou was lower than that from Changchun and Chengdu (*p* < 0.05). In terms of the GMP content, the milk from Lanzhou demonstrated a higher content than that from Tianjin, Chengdu, and Guangzhou (*p* < 0.05). The milk from Lanzhou had a higher AMP content than that from Changchun, Tianjin, Chengdu, and Guangzhou (*p* < 0.05); Shanghai was consistent with Lanzhou (*p* < 0.05).

**Figure 3:**
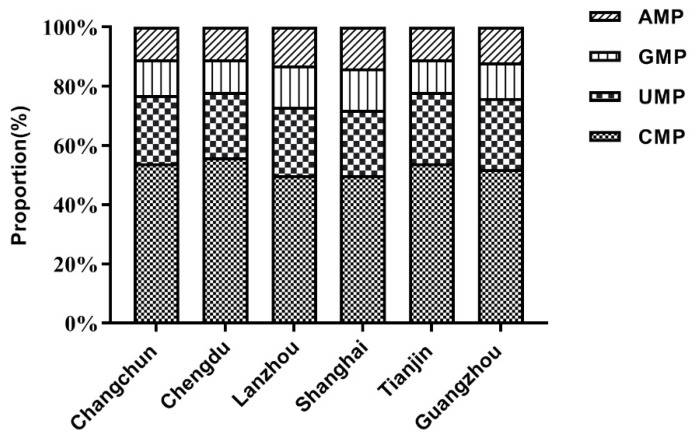
The proportion of total potential sources of nucleotides in the 6 regions. CMP, Cytidine monophosphate; UMP, uridine monophosphate; GMP, guanosine monophosphate; AMP, adenosine monophosphates.

The proportional composition of TPAN in the mature milk from the six regions is shown in Table 4 and Figure 3. CMP was the predominant nucleotide in the mature milk of the lactating mothers in the six regions, which accounted for 46.3–58.4% of TPAN. The statistical analysis showed that there was a significant difference in the TPAN composition of the milk from the different regions (*p* < 0.05). The proportions of CMP in the mature milk from Lanzhou and Shanghai were lower than that from Changchun, Chengdu, Tianjin, and Guangzhou (*p* < 0.05). In addition, the milk from Chengdu also had a higher CMP proportion than that from Changchun, Tianjin, and Guangzhou (*p* < 0.05). The proportion of UMP in the mature milk from Chengdu was lower than that from Tianjin and Guangzhou (*p* < 0.05). In terms of the GMP proportion, the milk from Lanzhou and Shanghai demonstrated higher proportions than that from Chengdu and Tianjin (*p* < 0.05). The proportion of GMP in the milk from Lanzhou was also higher than that from Changchun and Guangzhou (*p* < 0.05). The milk from Lanzhou and Shanghai had higher AMP proportions than that from Changchun, Chengdu, Tianjin, and Guangzhou (*p* < 0.05).

### 3.4. Correlations between Basic Demographic Characteristics and Nucleotides Content of Breast Milk

**Figure 4:**
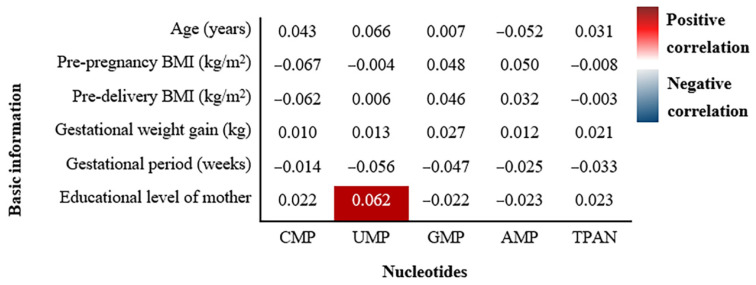
Correlations of basic demographic characteristics and TPAN contents in breast milk. The correlation coefficients (r) were shown in the graph.

The correlations between the basic demographic characteristics of the mothers and the TPAN content of their breast milk are shown in Figure 4. The TPAN content of the breast milk was not related to the basic maternal demographic characteristics, such as age, pre-pregnancy BMI, pre-delivery BMI, gestational weight gain, and gestational period. However, the UMP content of the breast milk was positively related to the educational level of the mother (r = 0.062, *p* = 0.048).

## 4. Discussion

To our knowledge, this is the first large-scale multicenter study in China to report the concentration and composition of TPAN in Chinese mature milk. Our study also analyzed the impact of demographic characteristics on the TPAN concentration of breast milk. This study expanded our knowledge of the nutrient content of Chinese breast milk and provided a scientific basis for nucleotide fortification of infant formula. 

The TPAN concentrations of Chinese mature breast milk notably varied among individuals. The difference in the TPAN concentrations of the breast milk was up to 10-fold, ranging from 11.61 to 111.09 mg/L. A similar result was reported in the study by Leach et al. [22], in which the TPAN concentration of European breast milk varied widely. A possible explanation could be the variable dietary protein intake in different regions. Studies have shown that consuming exogenous nucleic acids can increase nucleic acid synthesis through the salvage pathway [24]. Furthermore, nucleic acids in food are mostly combined with protein as a nuclear protein, which is more abundant in high-protein foods, such as aquatic food, red meat, and soy products [25]; thus, individual protein consumption may be responsible for the observed difference. Our results also showed that the TPAN concentration was higher in the breast milk of the participants from the traditionally high-protein dietary intake regions of China (such as Lanzhou and Changchun), although the difference was not significant.

The median 5′-MNT concentration of the mature milk was only 18.38% of the total TPAN concentration, which may underestimate the real demands of the infants. This finding was consistent with the European breast milk levels reported by Leach et al. [22]. Most studies have reported levels of monophosphate nucleotides in breast milk, as in most countries, nucleotide fortification of infant formula is achieved by adding monophosphate nucleotides [18,19,26]. However, the concentration of free monophosphate nucleotides in milk was affected by the pretreatment methods. The results of the study by Mateos-Vivass et al. showed that the monophosphate nucleotides content of breast milk samples treated by pasteurization was higher than in the control group [13]. The increase in free monophosphate nucleotides may have been due to the fact that the nucleoside polymers and adducts were hydrolyzed when the temperature was increased during the pasteurization step. Therefore, in our study, nucleoside polymers, adducts, and free nucleotides were hydrolyzed to the simplest form of nucleosides, and all of the nucleosides were expressed by TPAN, as to avoid the mutual transformation of nucleotides from different sources. TPAN could reflect the total nucleotide levels in breast milk better than the free monophosphate nucleotides. Accordingly, for the future development of infant formulas, the descriptive concentration of TPAN, as well as the detection methods of TPAN, can be used for detection and product label surveillance.

The TPAN in Chinese mature breast milk samples was mainly composed of the following four types of nucleotides: CMP, UMP, GMP, and AMP. Some studies have also reported the IMP content of breast milk [21,27], but IMP was not detected in our study. The detection of IMP is caused by the hydrolysis of adenosine deaminase in breast milk, which converts AMP into IMP [28]. In our study, method optimization was implemented to fully deactivate this enzyme in order to prevent AMP conversion; therefore, only the four nucleotides CMP, UMP, GMP, and AMP were detected in all samples. CMP was the predominant nucleotides type in the breast milk samples. The average proportions of CMP, UMP, GMP, and AMP were approximately 52.9%, 21.9%, 11.7%, and 11.6%, respectively. These results were similar to the findings of Leach et al. [22] and Tressler et al. [23]. Furthermore, the results demonstrated that, even with a large variance in TPAN content among populations, pyrimidine nucleotides occupied a dominant position in the breast milk of lactating mother from six regions in China. This indicates that the relatively consistent composition of TPAN may be linked to the unique needs of infancy, which requires further exploration.

There were no differences in the TPAN and UMP concentrations of the mature milk samples among the different regions; however, there were significant differences in the levels of the other three nucleotides. Although the CMP content and proportion were different in the six regions, the content and proportion of CMP were dominant in all of the regions. Purine nucleotide concentrations and proportions of samples from Lanzhou and Shanghai were significantly higher than those from other regions. Liao et al. [27] found that a relatively high intake of high-protein beans and chicken soup may be linked to the high nucleotide content of breast milk. Lanzhou in China was known to have a high-protein dietary pattern, beef and mutton were an indispensable part of the everyday diet of its residents. Shanghai, as a representative of the metropolitan areas of China, had a lifestyle with relatively high living standards; consequently, lactating mothers may have paid more attention to their protein intake. The levels of purine nucleotides in the mature milk may be affected by the protein intake of the lactating mothers; however, no research has been conducted to confirm our deduction to date.

No correlation was found between the maternal demographic characteristics and the TPAN content of the breast milk. The results showed that age, pre-gestational BMI, pre-delivery BMI, gestational weight gain, gestational period, parity, and delivery mode did not affect the TPAN content of the breast milk. The participants with a higher education level demonstrated higher UMP levels in their breast milk than those with a lower education level. These differences might be attributed to different lifestyles (physical activity, diet) and health care among the low- and high-education level participants. A study found that less educated women consumed a significantly lower amount of dairy products and salted cereals during the postpartum period [29]. Previous studies have shown that UMP was the predominant nucleotide in bovine colostrum [17]. The higher UMP content in the breast milk of the highly educated lactating mothers might be related to the intake of dairy. In previous studies, the relationship of macronutrients and fat-soluble vitamins in breast milk with maternal demographic characteristics and dietary habits has been systematically examined [30,31,32]. However, the TPAN concentration of breast milk in relation to the maternal demographic characteristics and dietary habits has not been reported, and needs to be further explored. Furthermore, nucleotides are known to support the growth of the gastrointestinal and immune systems in infants [6] and play an important role in promoting the catch-up growth of low birth weight (LBW) and preterm infants [33,34]. Hence, it is worth further investigating the role of nucleotides in the growth of LBW and preterm infants.

The regulatory allowable nucleotides fortification level in infant formula is varied world wide. For China, locally, the approved fortification range is 0.12~0.58 g/kg, which approximately might be converted to 15–73 mg/L in a reconstituted product. This fortification limit could be referred to the Chinese breast milk level, but the regulatory limit was published more than 10 years ago, and the cited data could be even older. Therefore, our study results provide a periodical review on the Chinese breast milk level. The range of the TPAN level in breast milk could be a reference to the nucleotide fortification in infant formula, at the same time infant formula must refer to clinical evidence in order to ensure the optimal delivery of health benefits. Clinical studies have shown that 72 mg/L nucleotide supplementation effectively reduced the risk of diarrhea in infants, supported the maturation of T cells and immune cell profile, and enhanced the response of infants to Hemophilus influenza B and diphtheria humoral antibodies [24,35,36,37]. Our results revealed that the median TPAN level of Chinese mature milk was lower than 72 mg/L; nevertheless, it is worth noting that 72 mg/L was still within the range of the TPAN levels in Chinese mature milk. In addition, our data provide scientific rationale to enlarge the regulatory allowable range in order to ensure the fortification level in products can meet the efficient level of TPAN.

## 5. Conclusions

In our study, the content and composition of TPAN in the mature breast milk from six regions of China were measured for the first time. The individual differences in the TPAN content of mature breast milk were relatively large, and TPAN was mainly composed of four types of nucleotides (CMP, UMP, GMP, and AMP) with a specific ratio range. CMP was the predominant nucleotide among the four nucleotides in all of the samples, accounting for 52.9% of the total TPAN content. There was no significant difference in the TPAN content of the mature milk samples from the six regions. The proportion of the four nucleotides was significantly different in the six regions. The contents of the free 5′-MNT in the mature milk were a small part of the TPAN contents; free monophosphate nucleotides cannot meet the needs of infant growth and development. Regarding the scientific basis for the content and form of the nucleotide fortification in infant formula, not only does the TPAN content and composition of breast milk need to be considered, but also the dose–effect relationship between the fortification dose and potential health effects on infants.

## Figures and Tables

**Table 1:** Basic demographic characteristics of lactating mothers and respective newborns in six regions.

Characteristic	Changchun	Chengdu	Lanzhou	Shanghai	Tianjin	Guangzhou
N	110	104	92	100	111	114
Age (years)	30.62 ± 3.21	30.09 ± 3.53	29.00 ± 4.34	29.58 ± 3.53	29.95 ± 3.19	28.91 ± 2.99
BMI (kg/m^2^), Pre-gestation	21.7 ± 3.2	21.0 ± 2.7	21.6 ± 3.0	21.3 ± 3.0	22.2 ± 4.0	20.5 ± 3.5
BMI (kg/m^2^), Pre-delivery	28.0 ± 4.9	26.4 ± 2.9	27.2 ± 3.2	26.8 ± 3.1	27.6 ± 3.5	25.9 ± 3.7
Gestational weight gain (kg)	17.4 ± 5.7	13.8 ± 4.2	14.7 ± 4.4	14.5 ± 5.1	14.6 ± 4.9	13.9 ± 5.2
Gestational period (weeks)	38.9 ± 1.2	39.0 ± 1.1	38.8 ± 2.0	39.5 ± 1.0	39.3 ± 1.1	38.9 ± 3.8
First pregnancy	59 (53.6)	35 (34.2)	50 (54.4)	53 (53.0)	68 (61.3)	70 (61.4)
Parity	73 (66.4)	37 (35.6)	50 (54.4)	54 (54.0)	70 (63.1)	70 (61.4)
Vaginal delivery	35 (31.8)	31 (29.8)	46 (50.0)	70 (70.0)	79 (71.2)	90 (79.0)
Educational level						
High school or below	7 (6.4)	22 (21.2)	31 (33.7)	15 (15.0)	28 (25.2)	19 (16.7)
College	71 (64.6)	64 (61.5)	57 (62.0)	72 (72.0)	64 (57.7)	84 (73.7)
Master or above	32 (29.1)	18 (17.3)	4 (4.4)	13 (13.0)	19 (17.1)	11 (9.7)
Offspring gender						
Male	53 (48.2)	47 (45.2)	41 (44.6)	60 (60.0)	53 (47.8)	56 (49.1)
Female	57 (51.8)	57 (54.8)	59 (55.4)	40 (40.0)	58 (52.3)	58 (50.9)
The occupation of mother						
Housework	7 (6.4)	16 (15.4)	24 (26.1)	13 (13.0)	19 (17.3)	9 (7.9)
Civil servant	23 (21.1)	23 (22.1)	35 (38.0)	17 (17.0)	27 (24.6)	7 (6.1)
Company employee	21 (19.3)	40 (38.5)	14 (15.2)	60 (60.0)	47 (42.7)	55 (48.3)
Worker or other occupation	58 (53.2)	25 (24.0)	19 (20.7)	10 (10.0)	17 (15.5)	43 (37.7)

Data were presented as the mean ± SD/*n* (%). BMI, body mass index, was calculated as body weight divided by height squared (kg/m^2^).

**Table 2:** The TPAN and 5′-MNT contents in mature milk.

	TPAN (*n* = 631)				5′-MNT (*n* = 283)			
	Median (P25, P75) (mg/L)	Min (mg/L)	Max (mg/L)	Percentage (%)	Median (P25, P75) (mg/L)	Min (mg/L)	Max (mg/L)	Percentage (%)
CMP	22.84 (17.93, 27.95)	2.35	63.88	52.9	4.67 (3.33, 6.07)	0.51	14.78	61.2
UMP	9.37 (7.33, 12.50)	2.69	32.00	21.9	1.86 (0.96, 3.23)	0.12	14.73	25.3
GMP	4.86 (3.40, 6.68)	1.23	32.28	11.7	0.40 (0.28, 0.64)	0.06	6.74	4.0
AMP	4.80 (3.54, 6.62)	1.24	28.62	11.6	0.67 (0.33, 1.09)	0.03	6.54	8.4
Total	43.26 (35.28, 52.15)	11.61	111.09	100	7.95 (5.03,11.06)	0.90	25.35	100

CMP, Cytidine monophosphate; UMP, uridine monophosphate; GMP, guanosine monophosphate; AMP, adenosine monophosphates; 5′-MNT, free 5′-monophosphate nucleotides; TPAN, total potentially available nucleosides.

**Table 3:** Comparison of TPAN contents in the 6 regions [mg/L, Median (P25, P75)].

	Changchun	Chengdu	Lanzhou	Shanghai	Tianjin	Guangzhou	*p*
N	110	104	92	100	111	114	—
CMP	24.20 (19.54, 31.73) ^a^	24.51 (20.16, 29.02) ^a^	20.10 (14.93,25.98) ^b^	21.82 (16.4, 27.09) ^ab^	22.83 (18.73,27.18) ^ab^	22.53 (17.05, 27.15) ^ab^	<0.001
UMP	9.55 (7.54, 13.43)	8.56 (7.06, 10.84)	9.57 (7.72, 12.87)	9.12 (7.10, 12.64)	9.24 (7.62, 12.22)	9.94 (7.40, 12.74)	0.051
GMP	5.16 (3.47, 7.00) ^ab^	4.32 (3.38, 6.01) ^b^	6.36 (3.99, 10.47) ^a^	5.25 (3.68, 7.60) ^ab^	4.20 (2.90, 6.36) ^b^	4.46 (3.23, 6.57) ^b^	<0.001
AMP	4.82 (3.29, 6.28) ^a^	3.97 (3.15, 5.19) ^b^	7.40 (4.63, 10.75) ^c^	6.01 (4.80, 7.25) ^c^	4.38 (3.19, 5.84) ^ab^	4.38 (3.32, 5.95) ^ab^	<0.001
TPAN	47.01 (35.10, 54.61)	42.58 (36.67, 48.82)	45.36 (34.28, 57.08)	43.83 (35.75, 50.54)	41.02 (33.14, 51.10)	42.12 (34.91, 51.94)	0.218

CMP, Cytidine monophosphate; UMP, uridine monophosphate; GMP, guanosine monophosphate; AMP, adenosine monophosphates; TPAN, total potentially available nucleosides. ^a,b,c^ mean values within a row with unlike superscript letters were significantly different (*p* < 0.05).

**Table 4:** Comparison of TPAN compositions in the 6 regions (%).

	Changchun	Chengdu	Lanzhou	Shanghai	Tianjin	Guangzhou	*p*
CMP	54.7 ^a^	58.4 ^b^	46.3 ^c^	50.0 ^c^	53.6 ^a^	52.9 ^a^	<0.001
UMP	21.6 ^ab^	20.6 ^a^	21.5^ab^	21.1^ab^	22.5 ^b^	23.5 ^b^	<0.001
GMP	11.6 ^ab^	10.4 ^b^	14.8 ^c^	12.6 ^ac^	10.1 ^b^	11.1 ^ab^	<0.001
AMP	10.7 ^a^	9.7 ^a^	16.2 ^b^	15.0 ^b^	10.7 ^a^	10.6 ^a^	<0.001

CMP, Cytidine monophosphate; UMP, uridine monophosphate; GMP, guanosine monophosphate; AMP, adenosine monophosphates. ^a,b,c^ mean values within a row with unlike superscript letters were significantly different (*p* < 0.05).

## Data Availability

The datasets generated or analyzed during the current study are not publicly available due to the data management requirements of our institution but are available from the corresponding author upon reasonable request.
